# pH-sensitive charge-conversion cinnamaldehyde polymeric prodrug micelles for effective targeted chemotherapy of osteosarcoma *in vitro*

**DOI:** 10.3389/fchem.2023.1190596

**Published:** 2023-05-03

**Authors:** Jiapeng Deng, Su Liu, Guoqing Li, Yien Zheng, Weifei Zhang, Jianjing Lin, Fei Yu, Jian Weng, Peng Liu, Hui Zeng

**Affiliations:** ^1^ National and Local Joint Engineering Research Center of Orthopaedic Biomaterials, Peking University Shenzhen Hospital, Shenzhen, China; ^2^ Department of Bone and Joint Surgery, Peking University Shenzhen Hospital, Shenzhen, China; ^3^ Department of Sports Medicine and Rehabilitation, Peking University Shenzhen Hospital, Shenzhen, China

**Keywords:** micelles, charge-conversion, pH-sensitive, cinnamaldehyde prodrug, osteosarcoma targeting

## Abstract

**Introduction:** Chemotherapy is a common strategy for the treatment of osteosarcoma. However, its therapeutic efficacy is not ideal due to the low targeting, lowbioavailability, and high toxicity of chemotherapy drugs. Nanoparticles can improve the residence time of drugs at tumor sites through targeted delivery. This new technology can reduce the risk to patients and improve survival rates. To achieve this goal, we developed a pHsensitive charge-conversion polymeric micelle [mPEG-*b*-P(C7-*co*-CA) micelles] for osteosarcoma-targeted delivery of cinnamaldehyde (CA).

**Methods:** First, an amphiphilic cinnamaldehyde polymeric prodrug [mPEG-*b*-P(C7-*co*-CA)] was synthesized through Reversible Addition-Fragmentation Chain Transfer Polymerization (RAFT) polymerization and post-modification, and self-assembled into mPEG-*b*-P(C7-*co*-CA) micelles in an aqueous solution. The physical properties of mPEG-*b*-P(C7-*co*-CA) micelles, such as critical micelle concentration (CMC), size, appearance, and Zeta potential were characterized. The CA release curve of mPEG-*b*-P(C7-*co*-CA) micelles at pH 7.4, 6.5 and 4.0 was studied by dialysis method, then the targeting ability of mPEG-*b*-P(C7-*co*-CA) micelles to osteosarcoma 143B cells in acidic environment (pH 6.5) was explored by cellular uptakeassay. The antitumor effect of mPEG-*b*-P(C7-*co*-CA) micelles on 143B cells *in vitro* was studied by MTT method, and the level of reactive oxygen species (ROS) in 143B cells after mPEG-*b*-P(C7-*co*-CA) micelles treatment was detected. Finally, the effects of mPEG-*b*-P(C7-*co*-CA) micelles on the apoptosis of 143B cells were detected by flow cytometry and TUNEL assay.

**Results:** An amphiphilic cinnamaldehyde polymeric prodrug [mPEG-*b*-P(C7-*co*-CA)] was successfully synthesized and self-assembled into spheric micelles with a diameter of 227 nm. The CMC value of mPEG-*b*-P(C7-*co*-CA) micelles was 25.2 mg/L, and it showed a pH dependent release behavior of CA. mPEG-*b*-P(C7-*co*-CA) micelles can achieve chargeconversion from a neutral to a positive charge with decreasing pHs. This charge-conversion property allows mPEG-*b*-P(C7-*co*-CA) micelles to achieve 143B cell targeting at pH 6.5. In addition, mPEG-*b*-P(C7-*co*-CA) micelles present high antitumor efficacy and intracellular ROS generation at pH 6.5 which can induce 143B cell apoptosis.

**Discussion:** mPEG-*b*-P(C7-*co*-CA) micelles can achieve osteosarcoma targeting effectively and enhance the anti-osteosarcoma effect of cinnamaldehyde *in vitro*. This research provides a promising drug delivery system for clinical application and tumor treatment.

## 1 Introduction

Osteosarcoma is the most common type of bone tumor in children and adolescents. The 5-year survival rate of patients is 65%–70%, while in patients with metastatic or recurrent diseases, the survival rate decreases to about 20% (Camuzard et al., 2019; Roessner et al., 2021). Although chemotherapy is an important treatment for osteosarcoma, the low solubility, low bioavailability, and severe side effects of antitumor drugs limit the application of chemotherapy (Wang and Xue, 2018).

In recent decades, nanoparticles have received great attention as drug delivery systems, as they can improve drug solubility, reduce toxicity, and increase the accumulation of drugs at tumor sites by the enhancing permeability and retention (EPR) effect and targeting delivery (Witika et al., 2020). Thus, to improve the therapeutic efficacy and reduce side effects of chemotherapy, many nanomedicine systems have been developed to deliver drugs targeted against osteosarcoma (Low et al., 2014; Rudnick-Glick et al., 2016; Yin et al., 2016). However, most of them achieve targeted delivery by decorating bone-targeting groups, such as the phosphate group, on the surface of nanoparticles (Son and Kim, 2017), which leads to a high accumulation of drugs in bones and damage to normal bone tissues due to the strong interaction between phosphate groups and bones (Rotman et al., 2018). Thus, it is highly desirable to develop new strategies to achieve osteosarcoma targeting.

It has been reported that surface charge plays an important role in the interaction between nanoparticles and cells in physiological environments (Hu et al., 2019). Nanoparticles with a positive charge present a strong interaction with tumor cells and a short blood circulation time, whereas nanoparticles with a negative charge present a weak interaction with tumor cells and a long blood circulation time (Du et al., 2014; Bernkop-Schnürch, 2018). As the tumor microenvironment usually presents as slightly acidic, a variety of nanoparticles with 2-(hexamethyleneimino) ethanol (C7) groups have been fabricated to achieve tumor targeting by charge conversion (Wang et al., 2018; Jin et al., 2019; Sun et al., 2021). These systems usually present a neutral charge under physiological conditions to facilitate long blood circulation time, and their surface charge changes to highly positive in the acidic tumor environment for effective tumor targeting. However, few C7-based charge-conversion systems were fabricated for osteosarcoma targeting, even osteolysis caused by osteosarcoma leads to a lower pH value of the acidic environment of osteosarcoma (Wu et al., 2018; Wu et al., 2021). Thus, developing C7-based charge-conversion nanoparticles is promising for achieving osteosarcoma-specific targeting by enhancing the interaction of nanoparticles with osteosarcoma cells by changing the surface charges of nanoparticles from negative to positive at osteosarcoma sites.

**SCHEME 1:**
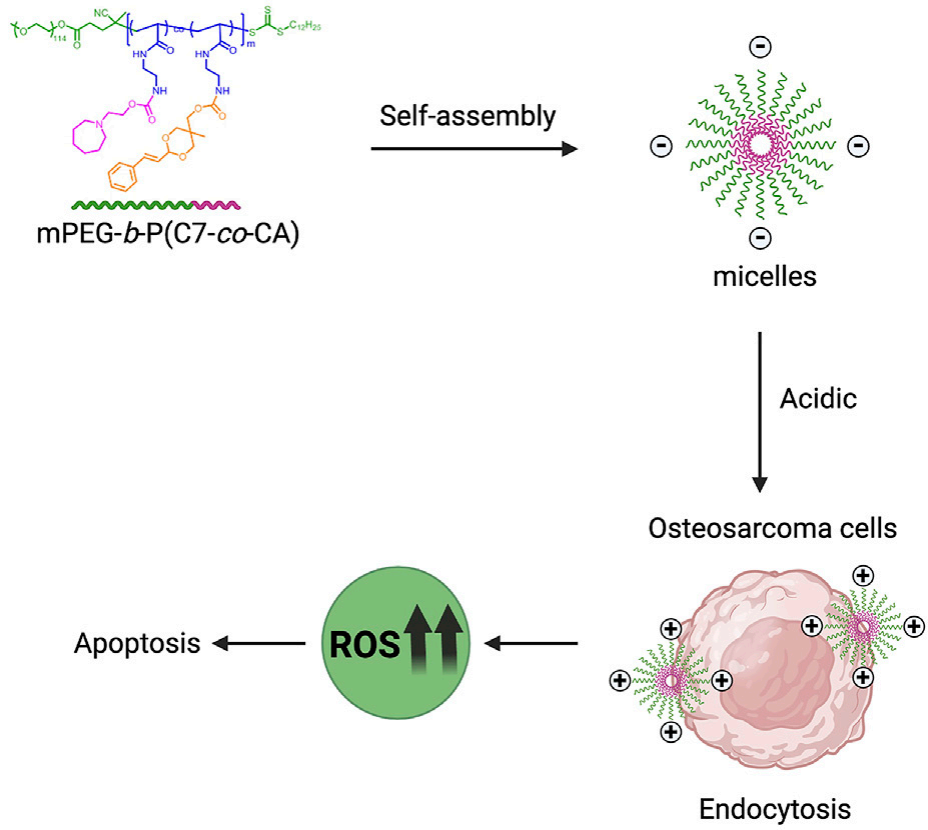
Schematic illustration of mPEG-*b*-P(C7-*co*-CA) micelles for effective chemotherapy of osteosarcoma through targeted delivery of cinnamaldehyde.

Cinnamaldehyde (CA) is the main component of cinnamon volatile oil and has many pharmacological effects, such as antibacterial, antioxidant, anti-inflammatory, hypoglycemic, and anti-tumor effects (Al Tbakhi et al., 2022). In recent years, studies have shown that CA can inhibit the proliferation and induce the apoptosis of osteosarcoma cells by regulating the Wnt/β-catenin and PI3K/AKT signaling pathways (Huang et al., 2020). In addition, it has been reported that CA can reduce the activity and protein level of the urokinase-type plasminogen activator, inhibit the migration and invasion ability of osteosarcoma cells, and significantly inhibit pulmonary metastasis of osteosarcoma in mice (Chu et al., 2022). Moreover, CA suppressed the self-renewal property and the expression of stemness genes in the 143B cells (Lin et al., 2022). CA is also known as an endogenous ROS generator to increase endogenous ROS production in tumor cells through mitochondrial dysfunction, thereby inhibiting tumor cell proliferation (Park and Baek, 2020; Zong et al., 2022). However, the disadvantages of CA are low solubility, high toxicity, and low bioavailability, which limit the application of CA as an antitumor drug (Ranjitkar et al., 2021). Thus, we developed a pH-sensitive charge-conversion cinnamaldehyde polymeric prodrug micelle [mPEG-*b*-P(C7-*co*-CA) micelles] for effective chemotherapy of osteosarcoma through targeted delivery, with the goal of promoting intracellular ROS generation and inducing osteosarcoma cell apoptosis (Scheme 1). It has been proven that the acid-labile acetal linkage has ideal stability at physiological pH and exhibits instability under acidic conditions (Feng et al., 2020). We conjugated CA to polymer through the acid-labile acetal linkage to enable the designed micelles to exhibit acid-responsive release behavior, allowing CA to be released from mPEG-*b*-P(C7-*co*-CA) micelles under acidic conditions.

## 2 Experiment

### 2.1 Reagents and materials

Polyethylene glycol monomethyl ether (mPEG-OH, M_W_ = 5,000), cinnamaldehyde , triethylamine (TEA), dicyclohexylcarbodiimide (DCC), 4-dimethylamino pyridine (DMAP), trifluoroacetic acid (TFA), dioxane, azodiisobutyronitrile (AIBN), and *N, N′*-carbonyldiimidazole (CDI) were purchased from Sigma-Aldrich Co., LLC (Shanghai, China). Also, 2-(hexamethyleneimino) ethanol (C7) was obtained from Shanghai Aladdin Biochemical Technology Co., Ltd. In addition, 3-(4,5-dimethylthiazole-2)-2,5-diphenyltetrazolium bromide (MTT) and 2′,7′-dichlorofluorescein diacetate (DCFH-DA) were purchased from Beyotime Biotechnology (Shanghai, China). Human osteosarcoma cell lines (143B) were purchased from the American Type Culture Collection (ATCC, United States).

### 2.2 Preparation of mPEG-*b*-P(C7-*co*-CA)

mPEG-CDTPA (0.5 g, 9.26 × 10^−2^ mmol), N-[2-(Boc-amino)ethyl]acrylamide (0.992 g, 4.63 mmol), and AIBN (3 mg, 1.83 × 10^−2^ mmol) were dissolved in 5 mL of dioxane. The solution was degassed with argon for 30 min and stirred for 14 h at 70°C. After the reaction, the solution was dropped into ether/hexane, and mPEG-*b*-P(Boc-AEAm) was obtained through centrifugation and drying under reduced pressure.

mPEG-*b*-P(Boc-AEAm) (1 g) was dissolved in 10 mL of DCM, and trifluoroacetic acid (4.68 mL) was added. After 3 h of reaction at room temperature (RT), mPEG-*b*-P (AEAm) was obtained through precipitation in excess ether, centrifugation, and drying under reduced pressure.

mPEG-*b*-P(AEAm) (0.1 g) was dissolved in 3 mL of *N,N*-dimethylformamide (DMF), and cinnamaldehyde-CDI (48.16 mg in 1 mL DMF, 0.15 mmol) and 0.127 mL of triethylamine were then added. After reacting for 14 h at 40°C, C7-CDI (69.6 mg in 1 mL DMF, 0.29 mmol) was added, and the solution was stirred for additional 14 h at 40°C. Finally, triethylamine (69.85 μL) and acetyl chloride (20.7 μL, 0.29 mmol) were added, and the reaction was carried out for another 5 h at 40°C. mPEG-*b*-P(C7-*co*-CA) was obtained by precipitation in excess ether, centrifugation, and drying under reduced pressure. mPEG-*b*-PC7A was prepared according to the same procedure without the addition of cinnamaldehyde-CDI.

### 2.3 Preparation of mPEG-*b*-P(C7-*co*-CA) micelles

mPEG-*b*-P(C7-*co*-CA) (5 mg) was dissolved in 1 mL of DMF and then dropped into 3 mL of DI water in 1 min while stirring. The mixture was stirred for another 30 min and then transferred into a dialysis tube (MW cutoff: 3,500) to dialyze against DI water for 1 day. mPEG-*b*-P(C7-*co*-CA) micelles were collected and stored at 4°C.

To prepare coumarin 6-loaded mPEG-*b*-P(C7-*co*-CA) micelles, mPEG-*b*-P(C7-*co*-CA) (5 mg) and coumarin 6 (0.05 mg) were dissolved in 1 mL of DMF and then dropped into 3 mL of DI water in 1 min while stirring. The mixture was stirred for another 30 min and transferred into a dialysis tube (MW cutoff: 3,500) to dialyze against DI water for 1 day. mPEG-*b*-P(C7-*co*-CA) micelles were collected and stored at 4°C. The entire procedure was carried out while avoiding light. mPEG-*b*-PC7A micelles were prepared according to the same procedure.

### 2.4 Characterization of mPEG-*b*-P(C7-*co*-CA) micelles

The critical micelle concentration (CMC) of mPEG-*b*-P(C7-*co*-CA) micelles was determined using Nile red as the probe. mPEG-*b*-P(C7-*co*-CA) micelle solutions with various concentrations between 0.3 mg/mL and 0.001 mg/mL were prepared, and the pH value was adjusted to 6.5 and 7.4; then, 1 μL of Nile red solution (0.4 mg/mL in acetone) was added to 1 mL of each mPEG-*b*-P(C7-*co*-CA) micelle solution. These samples were kept overnight for equilibrium. The fluorescence emission spectra were recorded from 570 to 750 nm using a λexc = 557 nm. The zeta potential and size change of the mPEG-*b*-P(C7-*co*-CA) micelles at different pH levels were determined using dynamic light scattering (DLS) (Malvern). The CA release profile from mPEG-*b*-P(C7-*co*-CA) micelles at different pHs was determined by dialysis method using a UV-Vis spectrometer (Cary 50, Varian, United States).

### 2.5 Targeting ability of mPEG-*b*-P(C7-*co*-CA) micelles against osteosarcoma at different pH levels

The 143B cells were seeded at 1 × 10^4^ cells per well in a 96-well plate and cultured overnight in Dulbecco’s modified Eagle medium (DMEM) (100 µL) containing 10% fetal bovine serum (FBS) at 37°C and 5% CO_2_. The pH of the medium was adjusted to 6.5 with 0.1 M HCl, and the pH value of the medium was measured using a pH meter (Mettler Toledo). The medium was then replaced with a serum-free medium containing coumarin 6-loaded mPEG-*b*-P(C7-*co*-CA) micelles (1 μg/mL of CA) at pH values 6.5 and 7.4, and the culture was continued for 3 h. Subsequently, the cells were washed with phosphate-buffered saline (PBS) and fixed with 4% paraformaldehyde at RT for 20 min. Finally, DAPI was used to stain the nucleus, and the fluorescence image was observed using a fluorescence microscope (DMI8 LEICA).

### 2.6 *In vitro* antitumor efficacy of mPEG-*b*-P(C7-*co*-CA) micelles

The 143B cells were seeded at 5 × 10^3^ cells per well in a 96-well plate in DMEM (100 µL) containing 10% FBS and incubated overnight at 37°C. The medium was then replaced with fresh DMEM (100 μL, pH 6.5 or 7.4) containing different concentrations of mPEG-*b*-P(C7-*co*-CA) micelles and CA. After 24 h of culture, the medium was replaced with fresh DMEM containing MTT (0.5 mg/mL), and the cells were cultured for another 4 h. After that, the medium was replaced with 100 µL of dimethyl sulfoxide in each well, and the medium was shaken at low speed in a shaker for 10 min to fully dissolve the crystals. The OD value at 595 nm was measured using a microplate reader (Multiskan GO). The data were expressed as average ±standard deviation (SD) (*n* = 3).

### 2.7 Detection of intracellular reactive oxygen species (ROS) levels

The 143B cells were seeded at 1 × 10^4^ cells per well in a 96-well plate in DMEM (100 µL) containing 10% FBS and incubated overnight at 37°C. The medium was then replaced with fresh DMEM (100 μL, pH 6.5 or 7.4) containing CA, mPEG-*b*-PC7A and mPEG-*b*-P(C7-*co*-CA) micelles (1 μg/mL of CA). The 143B cells were cultured for an additional 24 h at 37°C. The medium was removed, and the intracellular ROS level of the 143B cells was stained with 2′,7′-dichlorofluorescein diacetate (DCFH-DA) following the protocol. The fluorescence image was observed using a fluorescence microscope (DMI8 LEICA).

### 2.8 Cell apoptosis analysis by flow cytometry

The 143B cells were seeded at 1 × 10^6^ cells per well in a 6-well plate in DMEM (2 mL) containing 10% FBS and incubated overnight at 37°C. The medium was then replaced with fresh DMEM (2 mL, pH 6.5) containing mPEG-*b*-P(C7-*co*-CA) micelles (12 μg/mL of CA). The 143B cells were cultured at 37°C for 24 h, and the AnnexinV-FITC/PI Apoptosis Detection Kit (Dojindo Laboratories, Munich, Germany) was used, following the manufacturer’s protocols. Then, cell apoptosis was measured using a flow cytometry (Becton Dickinson, Mountain View, CA, United States). The results are presented as a percentage. The values indicated are the mean ± SD of three distinct experiments performed in triplicate.

### 2.9 TUNEL assay kit

The 143B cells were seeded at 1 × 10^4^ cells per well in a 24-well plate in DMEM (1 mL) containing 10% FBS and incubated overnight at 37°C. The medium was then replaced with fresh DMEM (1 mL, pH 6.5) containing mPEG-*b*-P(C7-*co*-CA) micelles (12 μg/mL of CA). After 24 h of incubation at 37°C, adherent cells were sequentially fixed with 4% paraformaldehyde (Beyotime Biotechnology) for 20 min and incubated with Triton X-100 (Beyotime Biotechnology) for 5 min at room temperature. Then, the TUNEL test solution was prepared according to the manufacturer’s protocols (Elabscience Biotechnology). The nuclei and TUNEL-positive cells were then observed under a fluorescence microscope (DMI8 LEICA), and the positive rate (TUNEL/DAPI) of three random fields was calculated.

### 2.10 Statistical analysis

The data are presented as means ± SD. Statistical analysis was performed by GraphPad Prism 8.0 (GraphPad Software Inc., San Diego, United States). Student’s t-test was used for differences between groups. Differences were statistically significant at the *p* < 0.05 level.

## 3 Results and discussion

### 3.1 Synthesis of mPEG-*b*-P(C7-*co*-CA)

**FIGURE 1:**
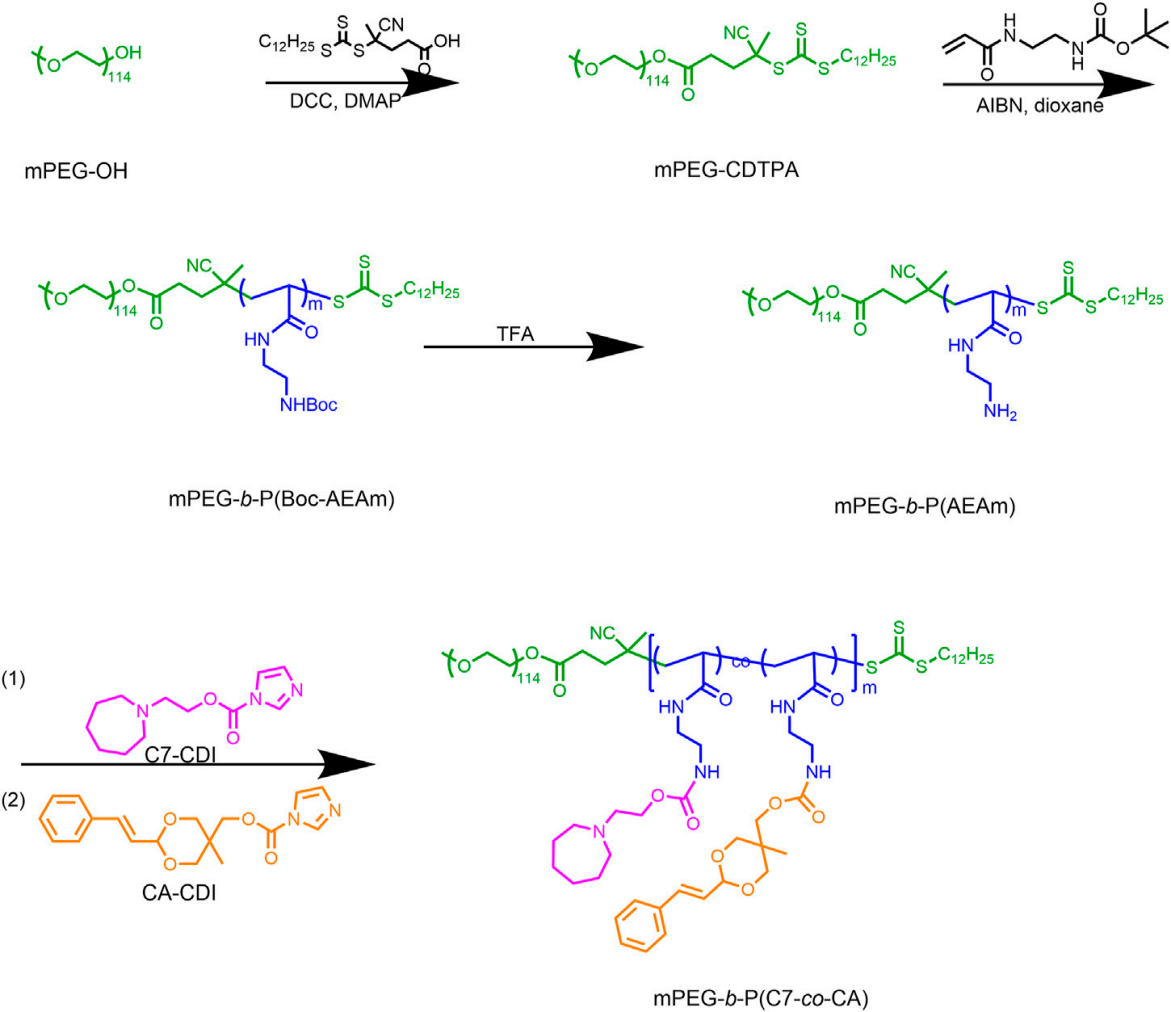
Synthetic procedure of mPEG-*b*-P(C7-*co*-CA).

**FIGURE 2:**
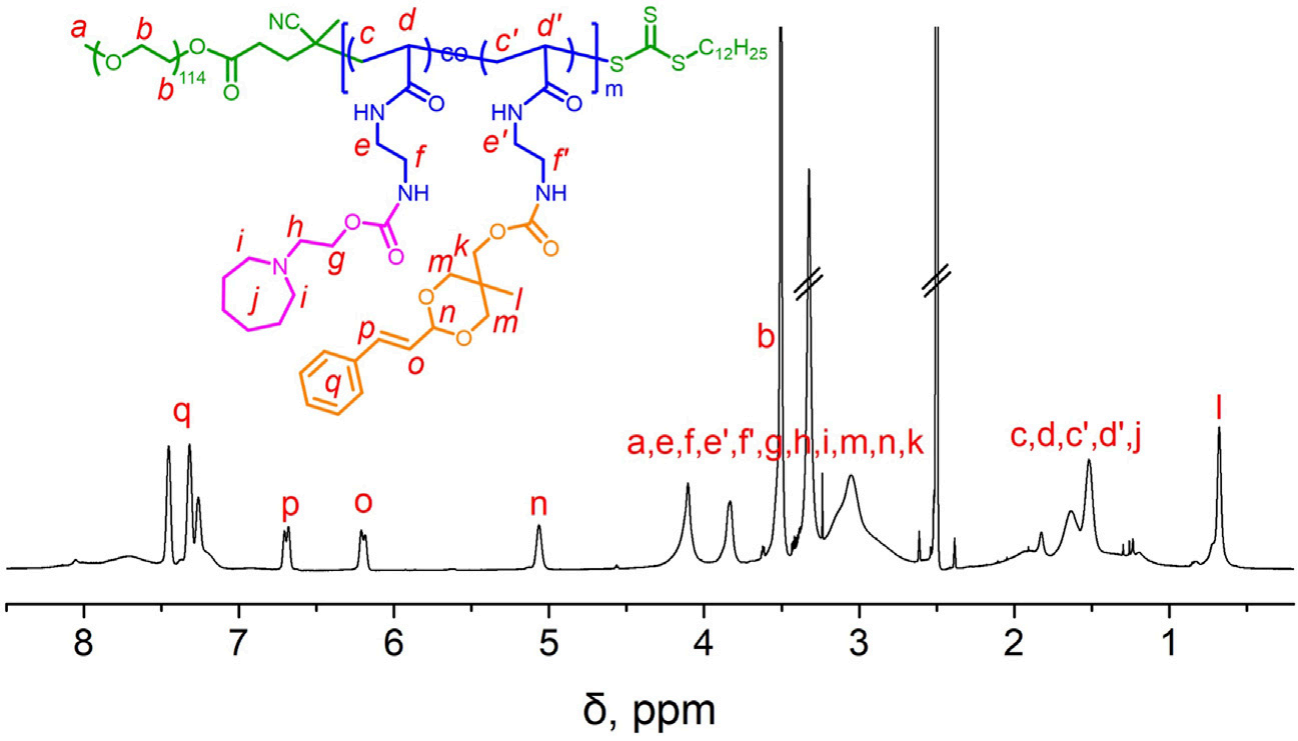
^1^H NMR spectrum of mPEG-*b*-P(C7-*co*-CA) in CDCl_3_.

To prepare the pH-sensitive charge-conversion-targeted nanomedicines, an amphiphilic cinnamaldehyde polymeric prodrug [mPEG-*b*-P(C7-*co*-CA)] was synthesized. The synthetic procedure is shown in Figure 1. First, mPEG-*b*-P(Boc-AEAm) was prepared using reversible addition–fragmentation chain transfer (RAFT) polymerization. The ^1^H NMR results (Supplementary Figures S1, S2) showed that the polymer was successfully synthesized, and the degree of polymerization was 48 (96% monomer conversion). The BOC group of mPEG-*b*-P(Boc-AEAm) was then removed with trifluoroacetic acid to obtain mPEG-*b*-P (AEAm). As shown in Supplementary Figure S2, the chemical shift at 1.43 ppm. belonging to the BOC group disappeared, and the ^1^H NMR results proved that all BOC groups were successfully removed. Finally, C7-CDI (Supplementary Figures S3) and cinnamaldehyde-CDI (Supplementary Figures S4, S5) were conjugated to mPEG-*b*-P (AEAm) to obtain the final amphiphilic cinnamaldehyde polymeric prodrug (mPEG-*b*-P(C7-*co*-CA)). The ^1^H NMR results (Figure 2) demonstrated the successful preparation of mPEG-*b*-P(C7-*co*-CA), and the amounts of cinnamaldehyde and C7 per mPEG-*b*-P(C7-*co*-CA) chain were determined to be 20 and 25, respectively. It means that 94% percent of AEA units were functionalized by CA and C7, and the ratio of C7 to CA in mPEG-*b*-P(C7-*co*-CA) was 5:4. In addition, the successful synthesis of mPEG-*b*-P(Boc-AEAm), mPEG-*b*-P (AEAm), and mPEG-*b*-P(C7-*co*-CA) was also confirmed by gel permeation chromatography (GPC) (Supplementary Figure S6), and the average molecular weights are summarized in Supplementary Table S1.

### 3.2 Preparation and characterization of mPEG-*b*-P(C7-*co*-CA) micelles

**FIGURE 3:**
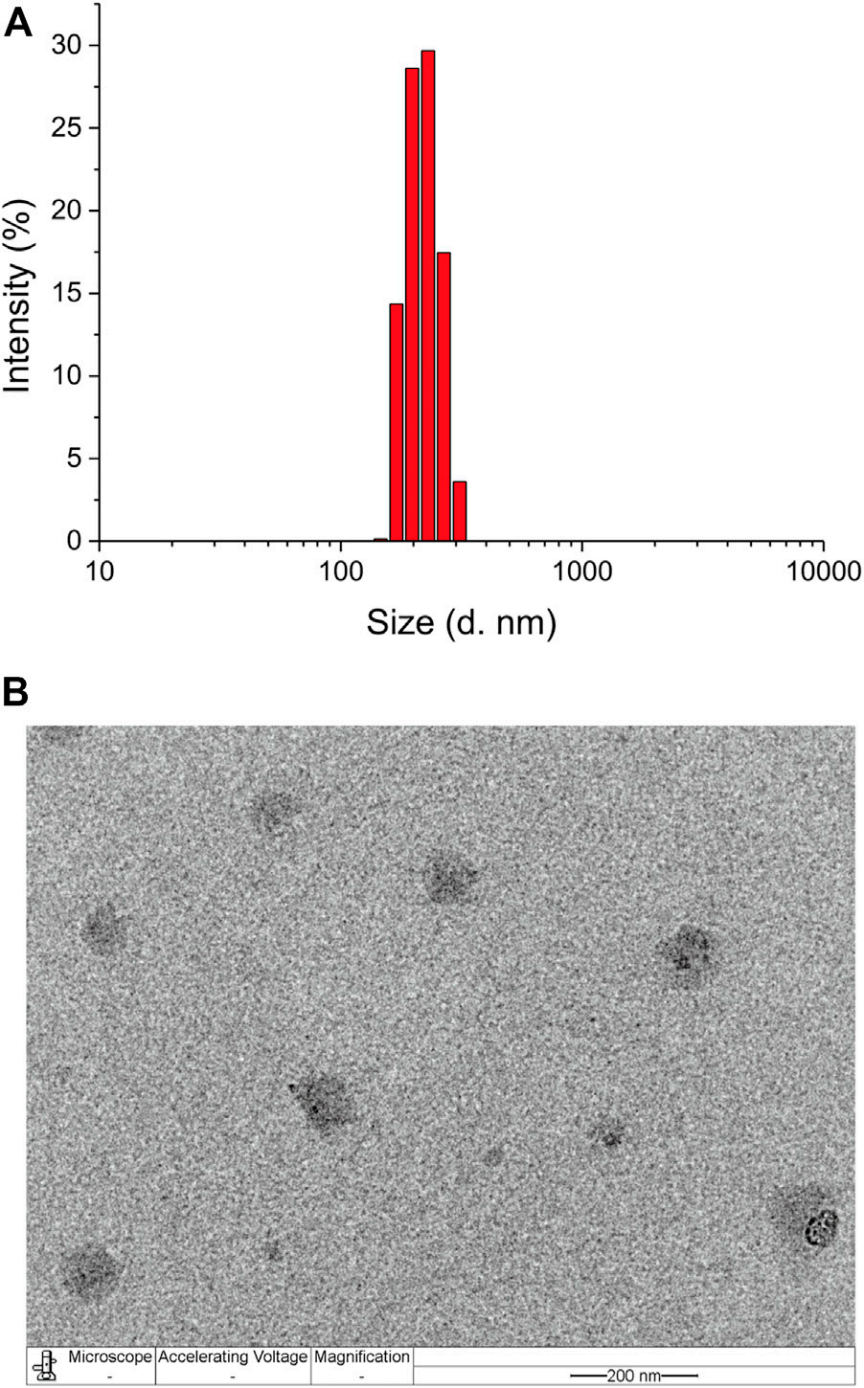
Size distribution of mPEG-*b*-P(C7-*co*-CA) micelles measured using DLS **(A)** and TEM **(B)** (scale bar 200 nm).

Amphiphilic polymers can self-assemble into micelles in aqueous solutions. Therefore, mPEG-*b*-P(C7-*co*-CA) micelles were prepared using the self-assembly method. The critical micelle concentration (CMC) of mPEG-*b*-P(C7-*co*-CA) micelles was determined utilizing Nile red as the fluorescent probe, and the measured CMC value was 25.2 mg/L, as shown in Supplementary Figures S7. The average size of the mPEG-*b*-P(C7-*co*-CA) micelles was determined to be around 227 nm with a low polydensity index (0.29) using DLS (Figure 3A), and the shape of the mPEG-*b*-P(C7-*co*-CA) micelles was spherical, as observed using TEM (Figure 3B). In addition, the mPEG-*b*-P(C7-*co*-CA) micelles have good reproducibility with a size fluctuation of ±16 nm.

**FIGURE 4:**
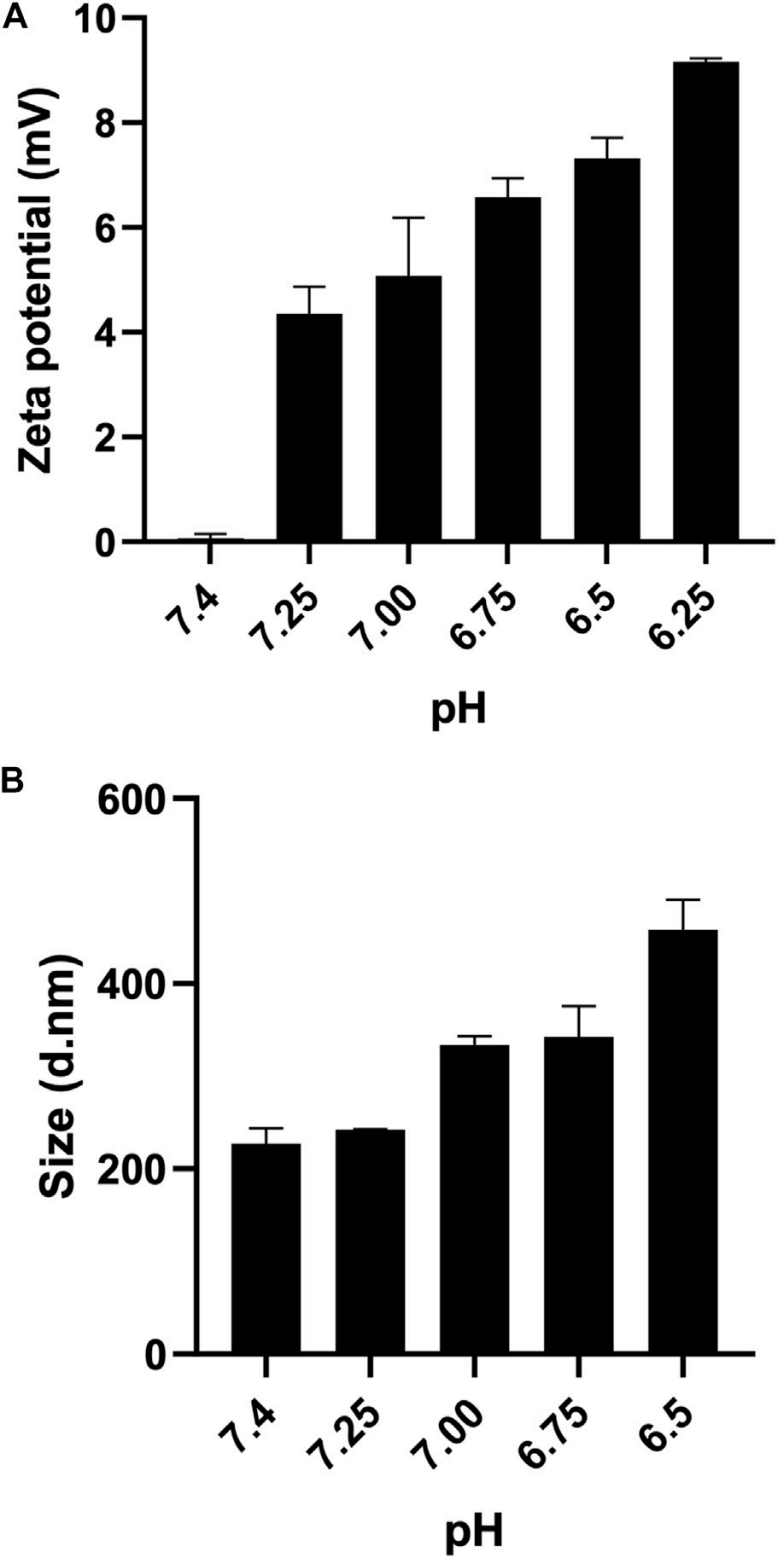
Zeta potential **(A)** and size **(B)** of mPEG-*b*-P(C7-*co*-CA) micelles at different pH levels.

To study the pH sensitivity of mPEG-*b*-P(C7-*co*-CA) micelles, the zeta potential of mPEG-*b*-P(C7-*co*-CA) micelles at different pHs (from 7.4 to 6.5) was measured. As shown in Figure 4A, the zeta potential of the mPEG-*b*-P(C7-*co*-CA) micelles was neutral at pH 7.4. However, the surface charge of mPEG-*b*-P(C7-*co*-CA) micelles increased dramatically with decreasing pH, and the zeta potential of the mPEG-*b*-P(C7-*co*-CA) micelles became +9.16 mV at pH 6.25. Meanwhile, the size changes of the mPEG-*b*-P(C7-*co*-CA) micelles at different pH levels were also evaluated. The results showed that the size of the mPEG-*b*-P(C7-*co*-CA) micelles increased gradually with decreasing pH (Figure 4B). The zeta potential and size changes were due to the protonation of the C7 group under acidic conditions. The C7 group protonation leads to an increase in the surface charge of mPEG-*b*-P(C7-*co*-CA) micelles, and the increase in the surface charge results in a size increase through electrostatic repulsion among the mPEG-*b*-P(C7-*co*-CA) chains. Moreover, the TEM image and CMC results of mPEG-*b*-P(C7-*co*-CA) micelles at pH 6.5 demonstrated that the mPEG-*b*-P(C7-*co*-CA) micelles undergo a slight shape change from sphere to worm, but they are still micelles with a CMC value of 23.5 mg/L under acidic conditions (Supplementary Figures S8). These results all indicate that mPEG-*b*-P(C7-*co*-CA) micelles can achieve charge conversion from physiological pH to acidic, facilitating the enhancement of the interaction between mPEG-*b*-P(C7-*co*-CA) micelles and osteosarcoma cells and achieving osteosarcoma targeting in an acidic environment.

**FIGURE 5:**
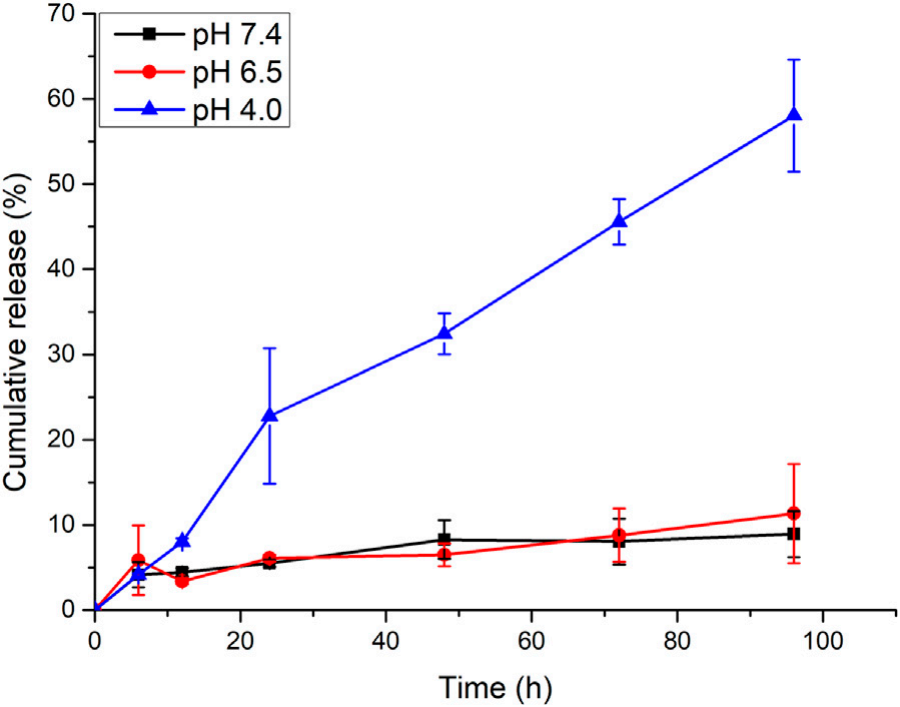
CA release profile from mPEG-*b*-P(C7-*co*-CA) micelles at different pHs.

In addition, the CA release profile from mPEG-*b*-P(C7-*co*-CA) micelles at pH 7.4, 6.5, and 4.0 was investigated by the dialysis method. mPEG-*b*-P(C7-*co*-CA) micelles present a pH-dependent CA release behavior. As shown in Figure 5, only about 11% of CA was released at pH 7.4 and 6.5 in 96 h, indicating that there was low CA leakage at physiological and extratumoral pH which can reduce the side effects of mPEG-*b*-P(C7-*co*-CA) micelles to normal tissues. However, approximately 59% of CA was released from mPEG-*b*-P(C7-*co*-CA) micelles at pH 4.0 in 96 h due to the breaking of acid-labile acetal linkage. It means that CA can be released rapidly from mPEG-*b*-P(C7-*co*-CA) micelles and achieve a therapeutic effect after mPEG-*b*-P(C7-*co*-CA) micelles entering osteosarcoma cells.

### 3.3 Cellular uptake of mPEG-*b*-P(C7-*co*-CA) micelles at different pH levels

**FIGURE 6:**
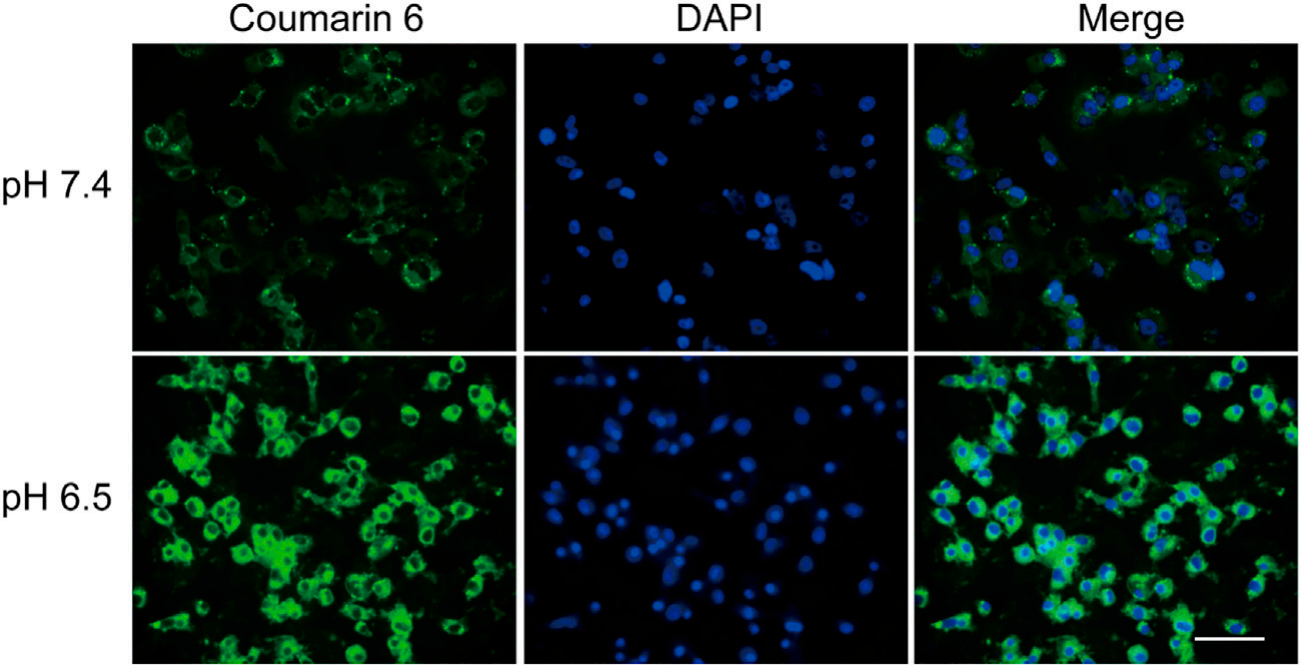
Fluorescence images of 143B cells treated with coumarin 6-loaded mPEG-*b*-P(C7-*co*-CA) micelles at pH 7.4 and 6.5 for 3 h (scale bar 100 µm).

As mPEG-*b*-P(C7-*co*-CA) micelles can achieve charge conversion with decreasing pH, the targeting ability of mPEG-*b*-P(C7-*co*-CA) micelles against osteosarcoma cells in a weakly acidic environment was verified using fluorescence microscopy. The 143B cells were selected as the model osteosarcoma cell. To achieve fluorescence tracing, coumarin 6-loaded mPEG-*b*-P(C7-*co*-CA) micelles were prepared. Coumarin 6-loaded mPEG-*b*-P(C7-*co*-CA) micelles were then co-incubated with 143B cells at pH values 7.4 and 6.5. After 3 h of incubation, the fluorescence signal of osteosarcoma was observed using fluorescence microscopy. As shown in Figure 6, 143B cells treated with coumarin 6-loaded mPEG-*b*-P(C7-*co*-CA) micelles at pH 6.5 showed stronger intracellular coumarin fluorescence compared with 143B cells cultured at pH 7.4. As the surface charge of mPEG-*b*-P(C7-*co*-CA) micelles can change from neutral to positive, mPEG-*b*-P(C7-*co*-CA) micelles have a stronger affinity to 143B cells with a negative surface charge in acidic environments. Therefore, the mPEG-*b*-P(C7-*co*-CA) micelles exhibited notable cellular uptake selectivity in different pH environments, enabling targeted cinnamaldehyde delivery to osteosarcoma.

### 3.4 *In vitro* antitumor efficacy of mPEG-*b*-P(C7-*co*-CA) micelles

**FIGURE 7:**
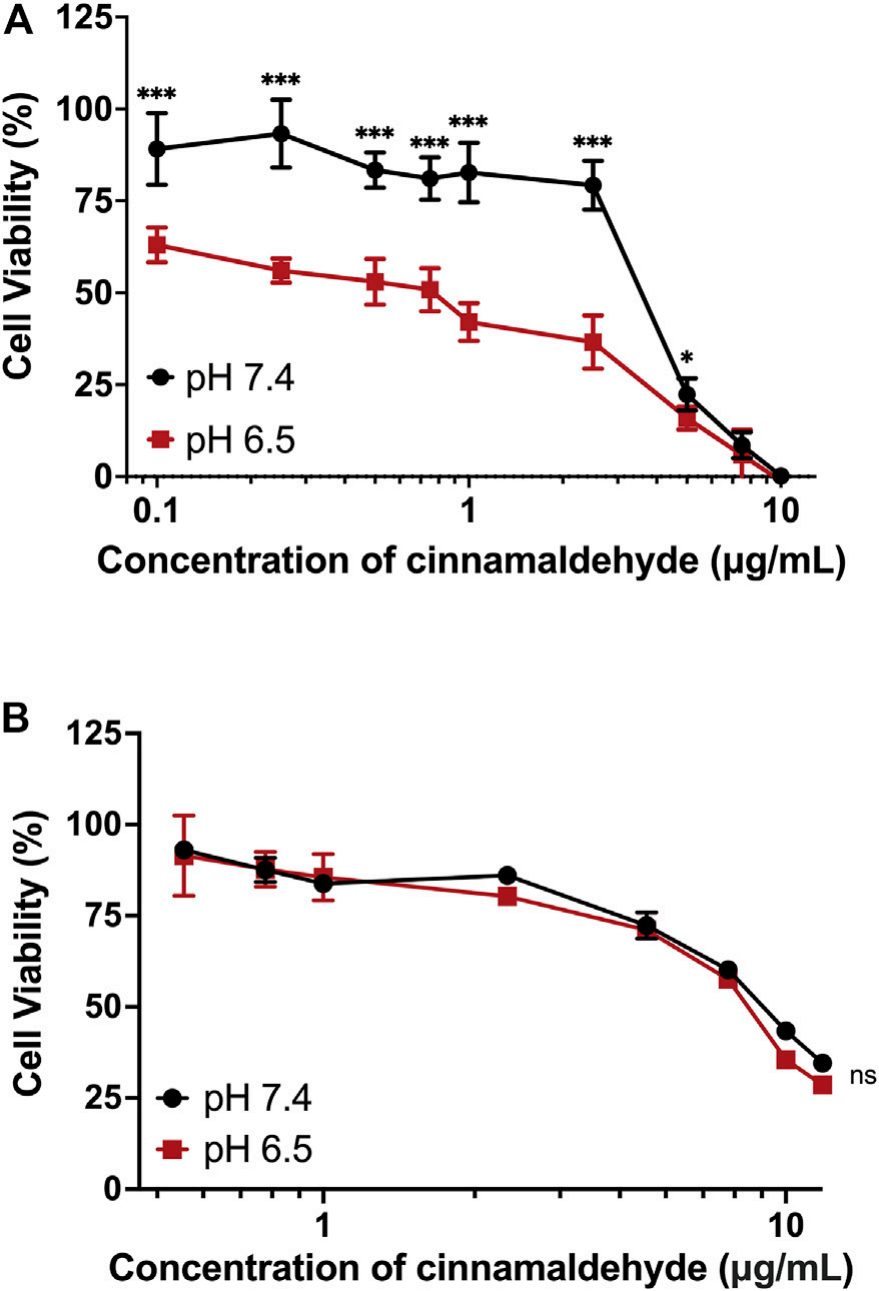
Cell viability of 143B cells treated with mPEG-*b*-P(C7-*co*-CA) micelles **(A)** and cinnamaldehyde **(B)** for 24 h at pH 7.4 and 6.5 (***p < 0.001, *p < 0.05, vs. the pH 7.4 group, *n* = 3).

It has been proven that mPEG-*b*-P(C7-*co*-CA) micelles can enter 143B cells more effectively in an acidic tumor microenvironment because of charge conversion. Therefore, the antitumor efficacy of mPEG-*b*-P(C7-*co*-CA) micelles against 143B cells at different pH levels (7.4 and 6.5) was investigated using the MTT method. As shown in Figure 7A, mPEG-*b*-P(C7-*co*-CA) micelles inhibited the proliferation of 143B cells more effectively at pH 6.5 than at pH 7.4, and mPEG-*b*-P(C7-*co*-CA) micelles at pH 6.5 presented higher antitumor efficacy. This is due to the stronger interaction of mPEG-*b*-P(C7-*co*-CA) micelles with 143B cells in a weak acidic environment, which achieves better tumor endocytosis, thereby exhibiting better antitumor efficacy. However, the antitumor efficacy of free cinnamaldehyde presents no notable difference at different pH levels (7.4 and 6.5) (Figure 7B).

### 3.5 Intracellular ROS levels of 143B cells

**FIGURE 8:**
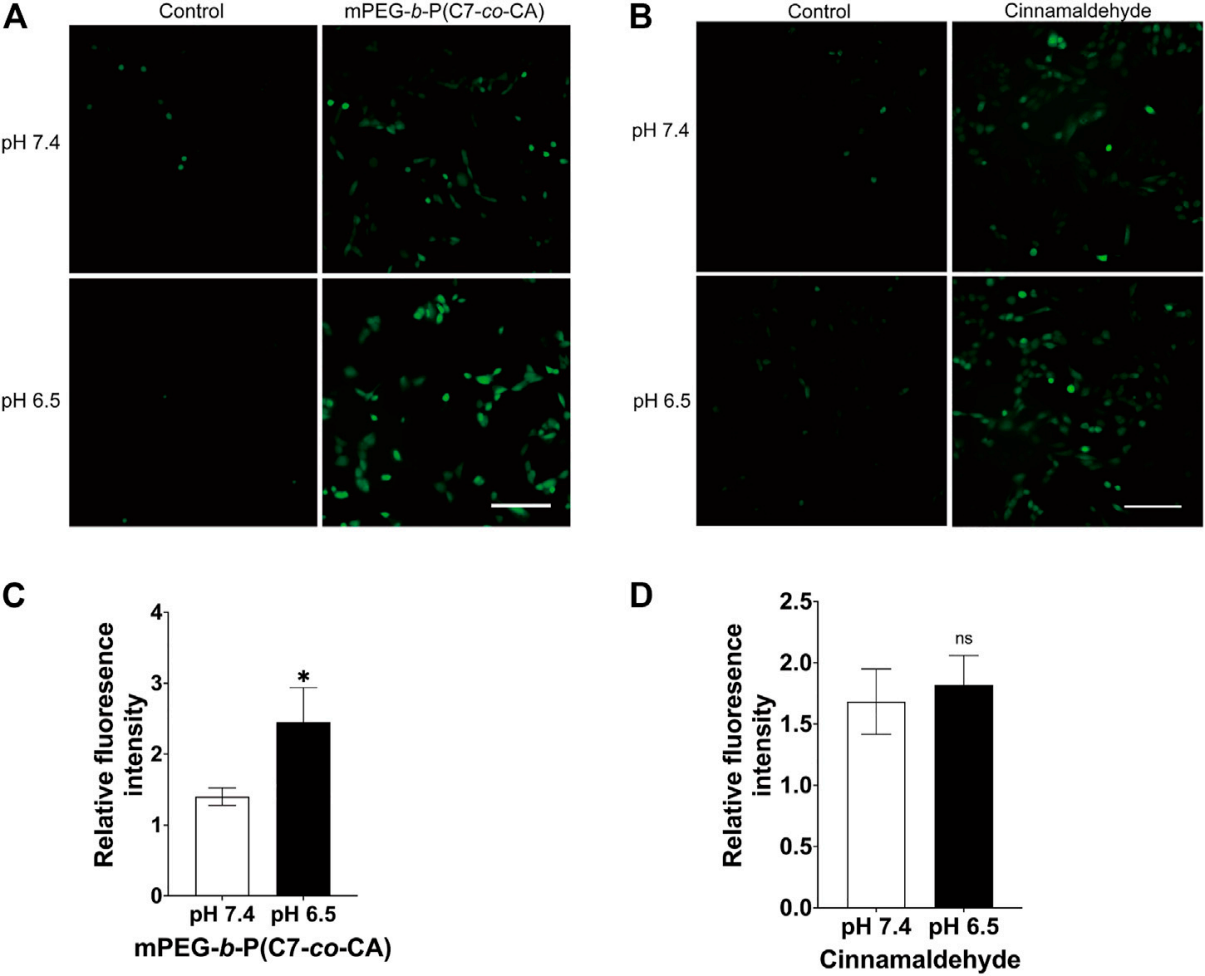
Intracellular ROS of 143B cells treated with mPEG-*b*-P(C7-*co*-CA) micelles **(A, C)** and cinnamaldehyde **(B, D)** at pH 7.4 and 6.5. DCFH-DA was used as the ROS probe (scale bar 200 μm, *p < 0.05, vs. the pH 7.4 group, *n* = 3).

Cinnamaldehyde can induce apoptosis and kill tumor cells by increasing the level of ROS in tumor cells (Dong et al., 2019). Therefore, the ROS level of 143B cells treated with mPEG-*b*-P(C7-*co*-CA) micelles and cinnamaldehyde under different pH conditions were studied using DCFH-DA as the ROS probe. Non-fluorescent DCFH-DA can be hydrolyzed to fluorescent DCF by ROS, and a green fluorescence signal was observed. In addition, the fluorescence intensity is proportional to the amount of ROS generated in cells (Eruslanov and Kusmartsev, 2010). As shown in Figure 8A, cells at pH 7.4 and 6.5 without mPEG-*b*-P(C7-*co*-CA) micelles present a low ROS signal. However, the 143B cells treated with mPEG-*b*-P(C7-*co*-CA) micelles at pH 7.4 showed a high ROS signal, and the strongest ROS signal was observed from 143B cells treated with mPEG-*b*-P(C7-*co*-CA) micelles at pH 6.5 (Figure 8C). Moreover, the ROS signal of 143B cells treated with CA and mPEG-*b*-PC7A micelles showed no notable difference under the conditions of pH 7.4 or 6.5 (Figures 8B, D and Supplementary Figure S9). These results indicate that mPEG-*b*-P(C7-*co*-CA) micelles can enter 143B cells more effectively, induce greater ROS generation, and achieve higher antitumor efficacy at pH 6.5.

### 3.6 Effect of mPEG-*b*-P(C7-*co*-CA) micelles on the apoptosis of 143B cells

**FIGURE 9:**
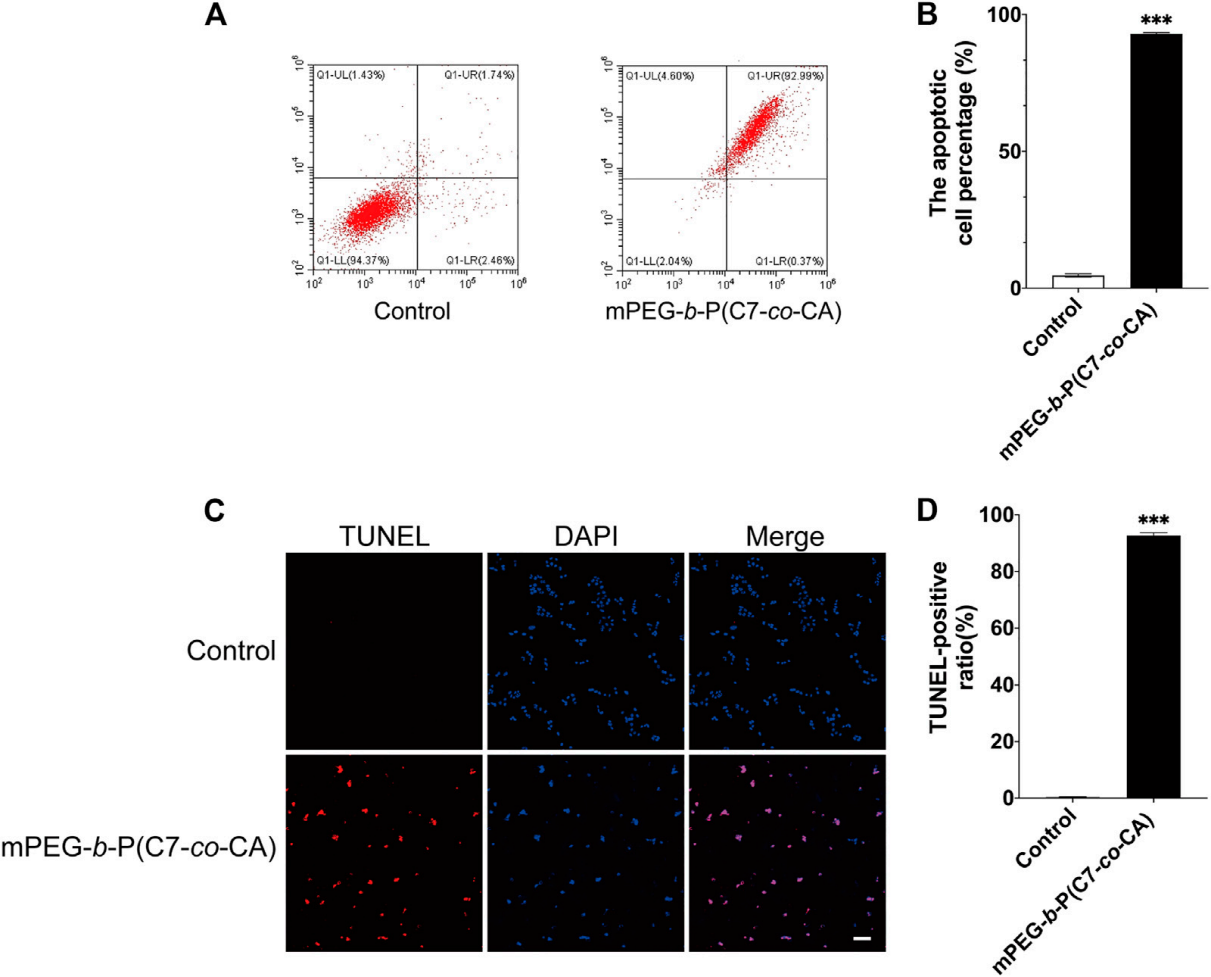
Effect of mPEG-*b*-P(C7-*co*-CA) micelles on the apoptosis of 143B cells was detected by flow cytometry **(A,B)** and TUNEL assays **(C, D)** (scale bar 100 μm, ***p < 0.001, vs. the control group, *n* = 3).

Studies have shown that the increase in ROS generation can cause the decrease in mitochondrial transmembrane potential, the release of cytochrome c, and then induce the apoptosis of tumor cells (Su et al., 2019). Therefore, we studied the effect of mPEG-*b*-P(C7-*co*-CA) micelles on apoptosis of 143B cells at pH 6.5 to simulate the acidic osteosarcoma environment. AnnexinV-FITC was used to quantify the effects of mPEG-*b*-P(C7-*co*-CA) micelles on 143B cell apoptosis. As shown in Figures 9A, B, mPEG-*b*-P(C7-*co*-CA) micelles increased the late apoptosis rate of cells compared with the control group. Moreover, TUNEL staining results also showed an increased apoptosis rate in the 143B cells treated with mPEG-*b*-P(C7-*co*-CA) micelles (Figures 9C, D). All these results indicated that mPEG-*b*-P(C7-*co*-CA) micelles could induce 143B cell apoptosis in the acidic tumor environment.

## 4 Conclusion

A novel pH-responsive cinnamaldehyde polymeric prodrug micelle for osteosarcoma-targeted delivery of cinnamaldehyde was successfully developed. mPEG-*b*-P(C7-*co*-CA) micelles have a diameter of 227 nm and a spherical shape. The surface charge of mPEG-*b*-P(C7-*co*-CA) micelles is neutral at physiological pH (7.4) and changes to positive at pH 6.5 (a weak acidic tumor microenvironment) through the protonation of the C7 group. This pH-induced charge conversion allowed mPEG-*b*-P(C7-*co*-CA) micelles to target the delivery of cinnamaldehyde to 143B cells at pH 6.5, and mPEG-*b*-P(C7-*co*-CA) micelles can induce 143B cell apoptosis more effectively by enhancing ROS generation in an acidic environment. All results proved that mPEG-*b*-P(C7-*co*-CA) micelles have great potential in the treatment of osteosarcoma.

## Data Availability

The original contributions presented in the study are included in the article/Supplementary Material; further inquiries can be directed to the corresponding authors.
