# Supplementary material for: pH-sensitive charge-conversion cinnamaldehyde polymeric prodrug micelles for effective targeted chemotherapy of osteosarcoma in vitro
Source: Front Chem. 2023 May 3;11:1190596. doi: 10.3389/fchem.2023.1190596 (PMC10188981; doi:10.3389/fchem.2023.1190596)
Supplement: Supplementary file 1 [file DataSheet1.docx]

Supplementary Material

pH-sensitive charge-conversion cinnamaldehyde polymeric prodrug micelles for effective targeted chemotherapy of osteosarcoma *in vitro*

Jiapeng Deng^†,1,2^, Su Liu^†,1,2^, Guoqing Li^†,1,2^, Yien Zheng^1,2^, Weifei Zhang^1,2^, Jianjing Lin^3^, Fei Yu^1,2^, Jian Weng^*,1,2^, Peng Liu^*,1,2^ and Hui Zeng^*,1,2^

^1^National & Local Joint Engineering Research Center of Orthopaedic Biomaterials, Peking University Shenzhen Hospital, Shenzhen, China

^2^Department of Bone & Joint Surgery, Peking University Shenzhen Hospital, Shenzhen, China

^3^Department of Sports Medicine and Rehabilitation, Peking University Shenzhen Hospital, Shenzhen, China

***Correspondence:** Jian Weng, [jweng@pku.edu.cn](mailto:jweng@pku.edu.cn); Peng Liu, [liupeng_polymer@126.com](mailto:liupeng_polymer@126.com); Hui Zeng, [zenghui_36@163.com](mailto:zenghui_36@163.com)
^†^These authors contributed equally to this work

# Synthesis of mPEG-CDTPA

mPEG-OH (4.35 g, 0.87 mmol), 4-cyano-4-[(dodecylsulfanylthiocarbonyl)sulfanyl]pentanoic acid (CDTPA, 0.6 g, 1.48 mmol) and 4-dimethylaminopyridine (DMAP, 36 mg, 0.29 mmol) were dissolved in 15 mL of dichloromethane, and *N,N′*-dicyclohexylcarbodiimide (DCC, 0.366 g, 1.78 mmol) was added. The reaction was allowed to proceed overnight at room temperature. The white precipitate was removed by filtration. mPEG-CDTPA was obtained by precipitation in excess diethyl ether, centrifugation, and drying under reduced pressure.

**Synthesis of C7-CDI**

**
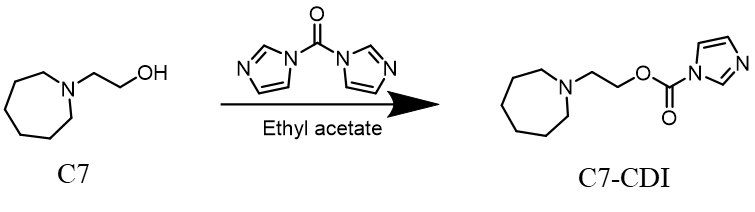
**

2-(Hexamethyleneimino)ethanol (C7, 1 g, 6.98 mmol) was dissolved in 40 mL of ethyl acetate, and *N, N'*-carbonyldiimidazole (CDI, 2.26 g, 13.9 mmol) was added. The reaction was allowed to proceed for 3 h at room temperature. The mixture was washed with water 3 times and dried over anhydrous MgSO_4_. The C7-CDI was obtained through filtration and evaporation of the solvent.

**Synthesis of cinnamaldehyde derivatives (compound 1)**

**
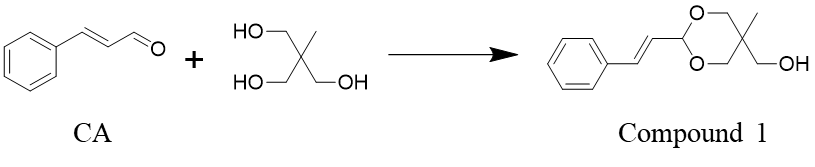
**

1,1,1-trimethylolethane (4.88 g, 40.62 mmol), dibutyltin dilaurate (DBTL, 100 μL) and cinnamaldehyde (CA, 5.7 mL, 45.11 mmol) were dissolved in 70 mL of tetrahydrofuran, then 40 mg of p-toluenesulfonic acid was added. The reaction was allowed to proceed for 3 h at 40℃. After the mixture was cooled, 1 mL of triethylamine was added to stop the reaction. The solvent was removed, and the crude product was purified via column chromatography to obtain the final compound 1.

**Preparation of cinnamaldehyde-CDI** (**CA-CDI)**

**
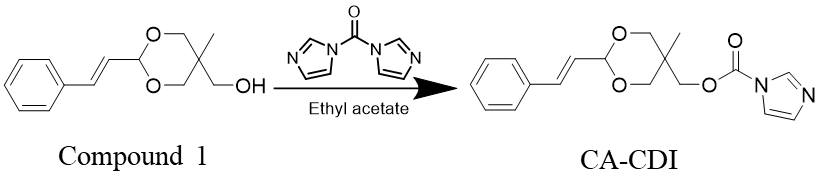
**

Compound 1 (0.5 g, 2.14 mmol) was dissolved in 40 mL of ethyl acetate, and 1.38 g (8.51 mmol) of CDI was added. The reaction was allowed to proceed for 1.5 h at room temperature. The mixture was washed with water 3 times and dried over anhydrous MgSO_4_. The CA-CDI was obtained through filtration and evaporation of the solvent.

# Supplementary Figures and Tables


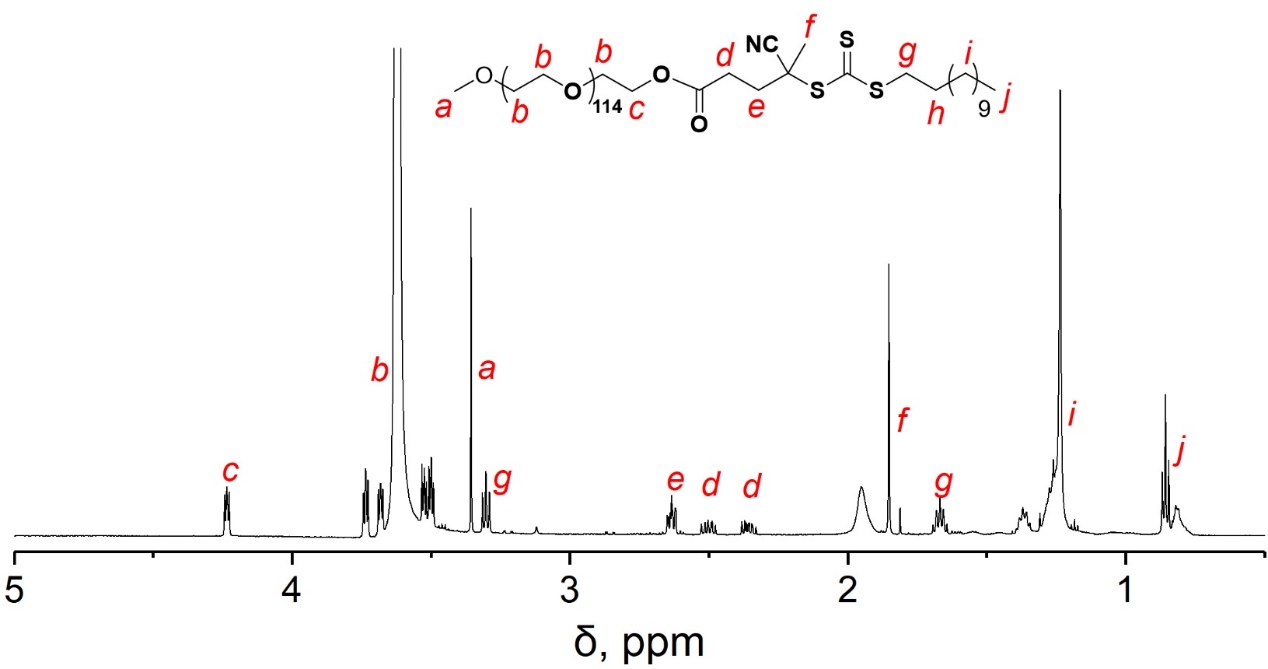


**FIGURE S1.** ^1^H NMR spectrum of mPEG-CDTPA in CDCl_3_.


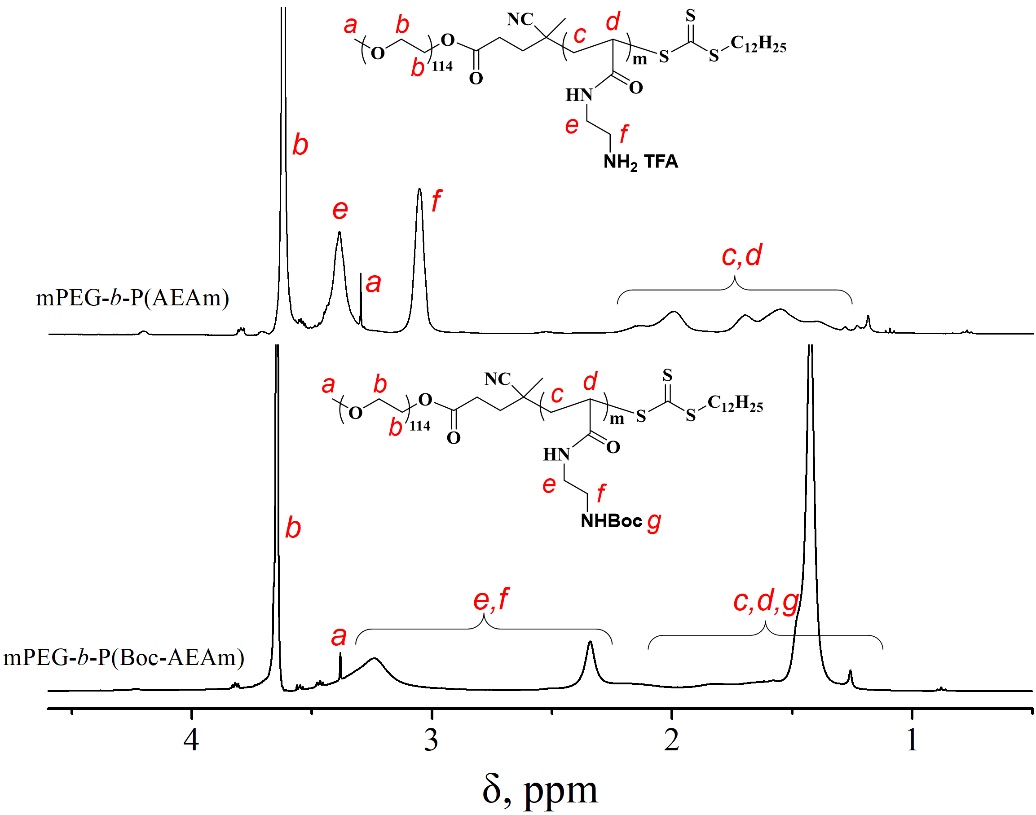


**FIGURE S2.** ^1^H NMR spectra of mPEG-*b*-P(Boc-AEAm) in CDCl_3_ and mPEG-*b*-P(AEAm) in D_2_O.


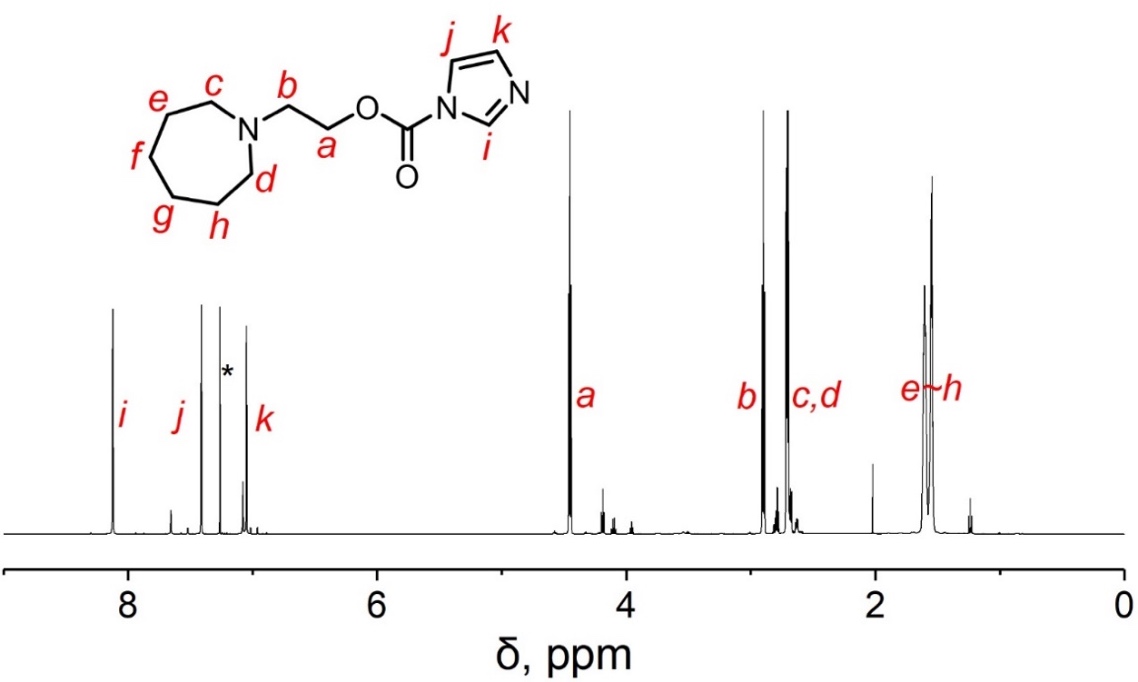


**FIGURE S3.** ^1^H NMR spectrum of C7-CDI in CDCl_3_.

**
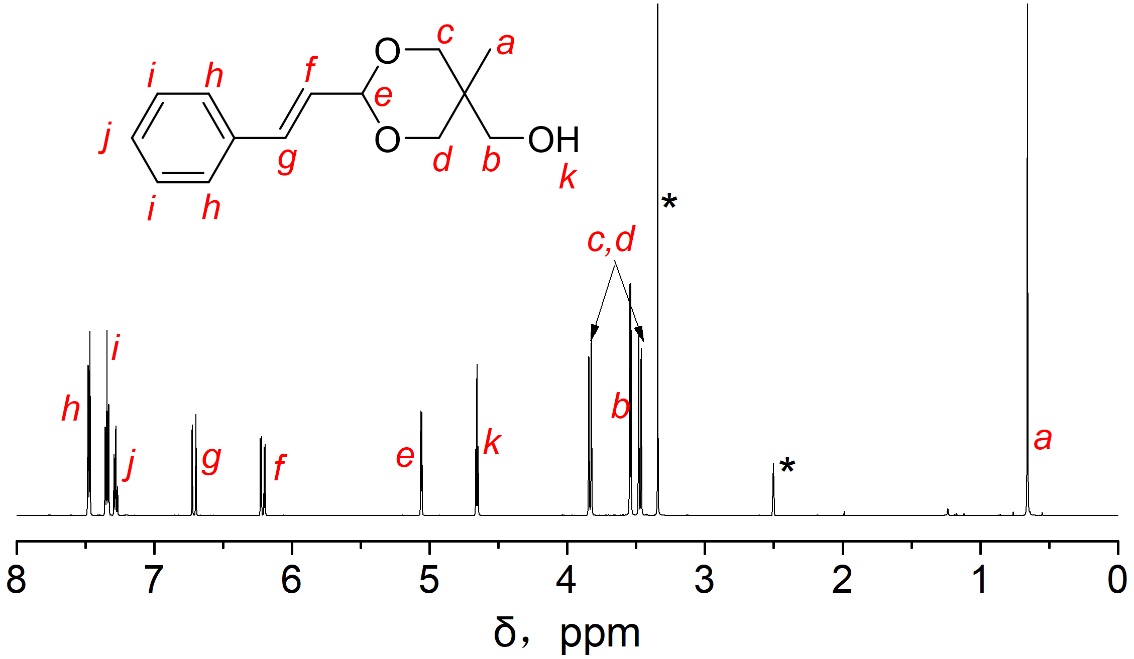
**

**FIGURE S4.** ^1^H NMR spectrum of compound 1 in DMSO-d_6_.


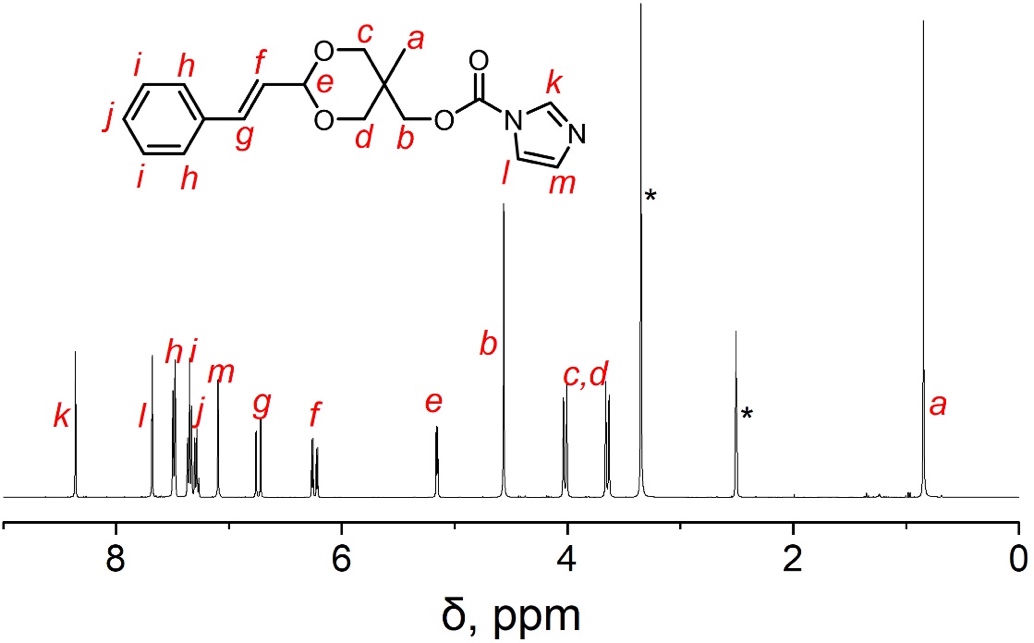


**FIGURE S5.** ^1^H NMR spectrum of CA-CDI in DMSO-d_6_.


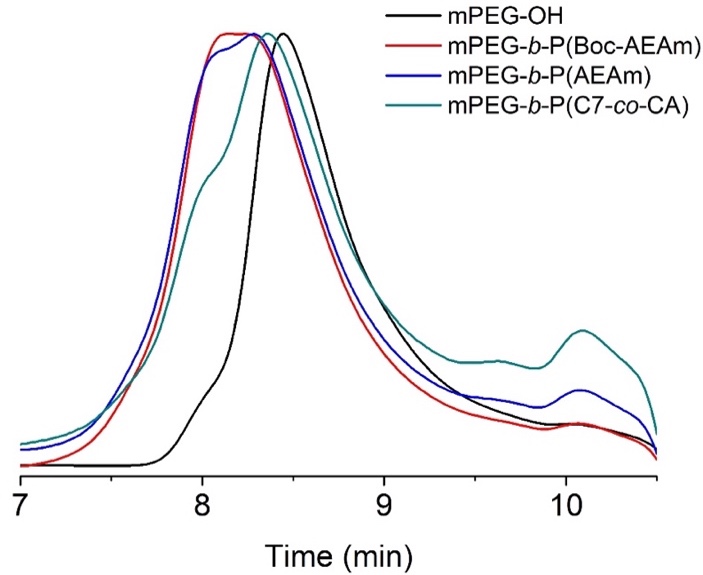


**FIGURE S6.** GPC traces of mPEG-OH, mPEG-*b*-P(Boc-AEAm), mPEG-*b*-P(AEAm) and mPEG-*b*-P(C7-*co*-CA) (Eluent: DMF, flow rate: 1 mL/min, standards: polystyrene).

**TABLE S1.** Molecular Weight and Polydispersity of the Polymers determined by GPC.

|  | Mn | Mw | Polydispersity |
| --- | --- | --- | --- |
| mPEG-OH | 6112 | 9207 | 1.50 |
| mPEG-*b*-P(Boc-AEAm) | 9661 | 18472 | 1.91 |
| mPEG-*b*-P(AEAm) | 10483 | 18841 | 1.79 |
| mPEG-*b*-P(C7-*co*-CA) | 9574 | 16266 | 1.69 |


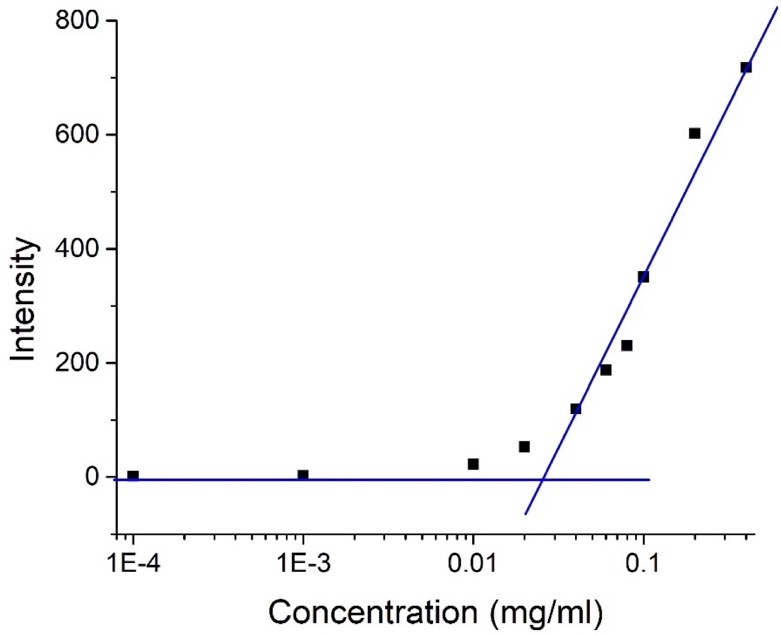


**FIGURE S7.** The CMC of mPEG-*b*-P(C7-*co*-CA) at pH 7.4 are determined utilizing Nile red as fluorescence probe.


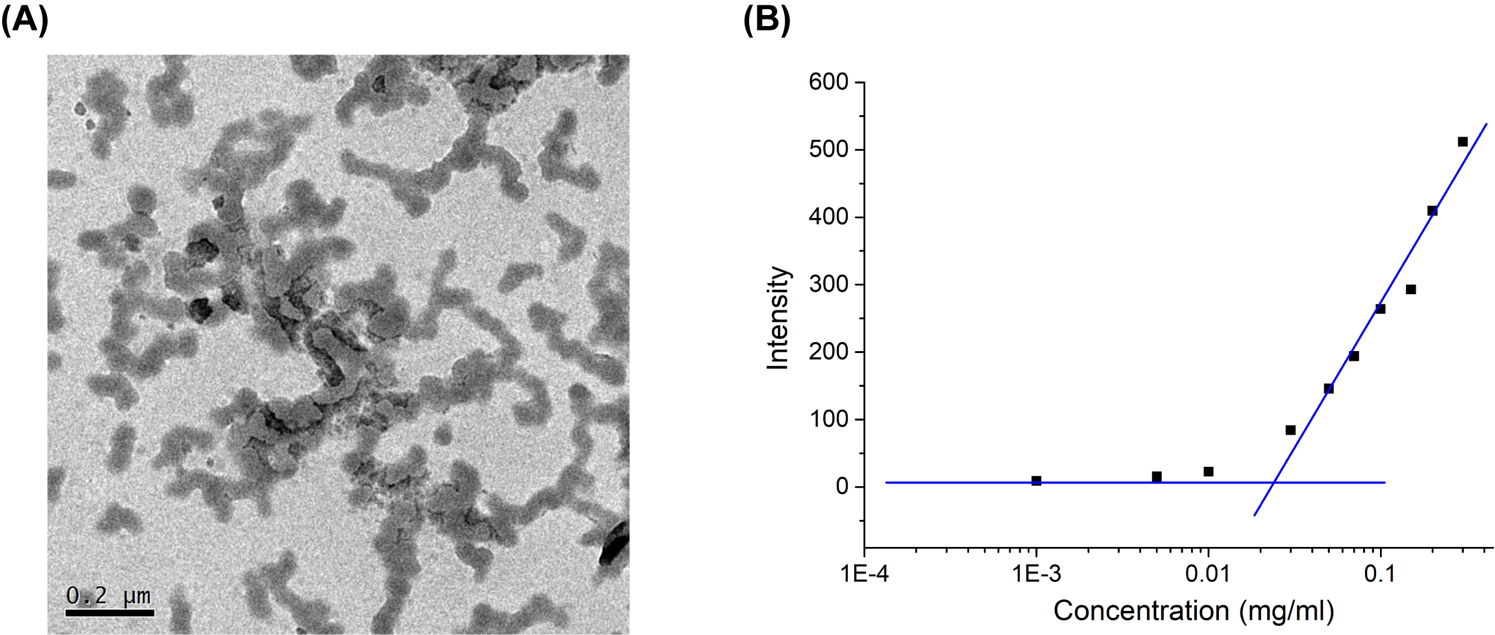


**FIGURE S8.**The TEM image of mPEG-*b*-P(C7-*co*-CA) micelles at pH 6.5 (A) (Scale bar 0.2 µm). The CMC of mPEG-*b*-P(C7-*co*-CA) at pH 6.5 determined utilizing Nile red as fluorescence probe (B).


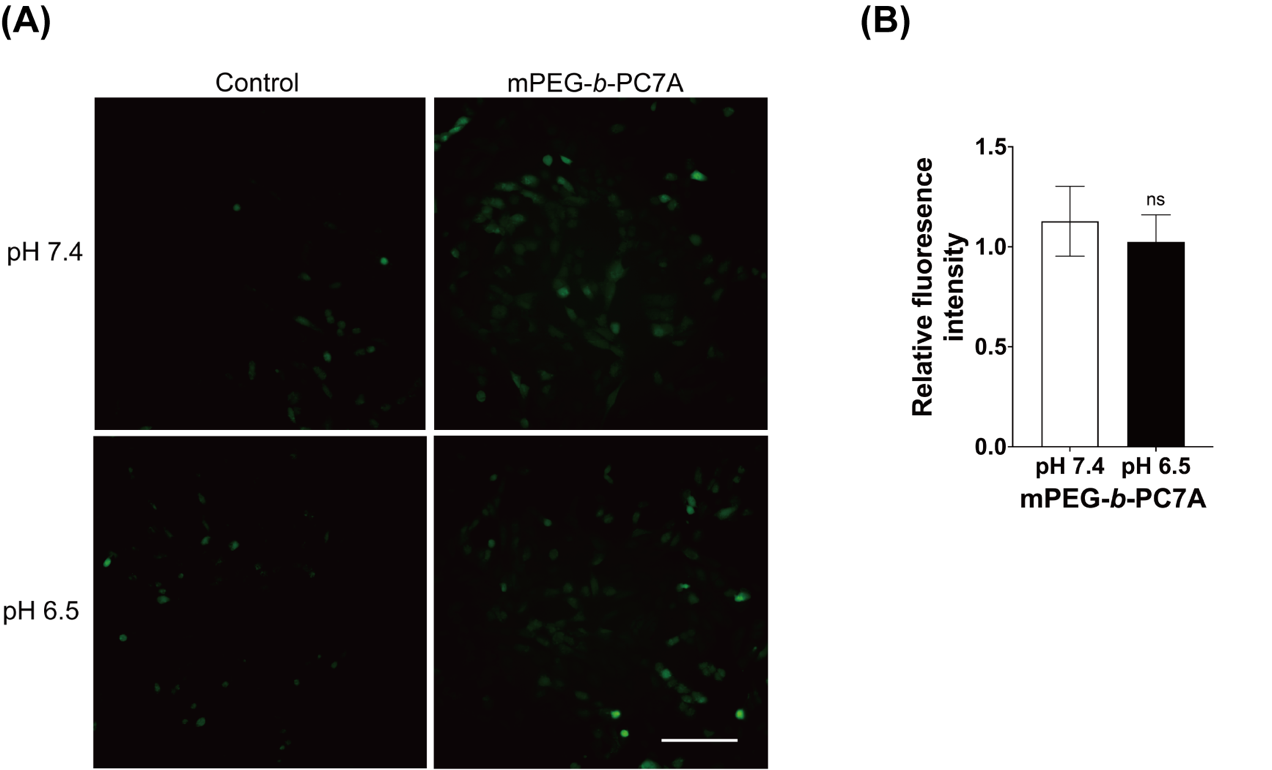


**FIGURE S9.** Intracellular ROS of 143B cells treated by mPEG-*b*-PC7A micelles (A and B) at pH 7.4 and 6.5. DCFH-DA was used as the ROS probe (Scale bar 200 µm, vs the pH 7.4 group, n=3).
